# ViralmiR: a support-vector-machine-based method for predicting viral microRNA precursors

**DOI:** 10.1186/1471-2105-16-S1-S9

**Published:** 2015-01-21

**Authors:** Kai-Yao Huang, Tzong-Yi Lee, Yu-Chuan Teng, Tzu-Hao Chang

**Affiliations:** 1 Department of Computer Science and Engineering, Yuan Ze University, Taoyuan 320, Taiwan; 2 Innovation Center for Big Data and Digital Convergence, Yuan Ze University, Taoyuan 320, Taiwan; 3 Graduate Institute of Biomedical Informatics, Taipei Medical University, Taipei 110, Taiwan

## Abstract

**Background:**

microRNAs (miRNAs) play a vital role in development, oncogenesis, and apoptosis by binding to mRNAs to regulate the posttranscriptional level of coding genes in mammals, plants, and insects. Recent studies have demonstrated that the expression of viral miRNAs is associated with the ability of the virus to infect a host. Identifying potential viral miRNAs from experimental sequence data is valuable for deciphering virus-host interactions. Thus far, a specific predictive model for viral miRNA identification has yet to be developed.

**Methods and results:**

Here, we present ViralmiR for identifying viral miRNA precursors on the basis of sequencing and structural information. We collected 263 experimentally validated miRNA precursors (pre-miRNAs) from 26 virus species and generated sequencing fragments from virus and human genomes as the negative dataset. Support vector machine and random forest models were established using 54 features from RNA sequences and secondary structural information. The results show that ViralmiR achieved a balanced accuracy higher than 83%, which is superior to that of previously developed tools for identifying pre-miRNAs.

**Conclusions:**

The easy-to-use ViralmiR web interface has been provided as a helpful resource for researchers to use in analyzing and deciphering virus-host interactions. The web interface of ViralmiR can be accessed at http://csb.cse.yzu.edu.tw/viralmir/.

## Introduction

microRNAs (miRNAs) are non-protein-coding RNAs that is approximately 22 nucleotides long, which results in the degradation of mRNAs by complementarily binding to the 3' untranslated regions of target genes. Recent studies have demonstrated that miRNAs play a vital role in development, oncogenesis, and apoptosis by binding to mRNAs to regulate the posttranscriptional level of coding genes in mammals, plants, and insects. In addition, miRNAs modulate viral existence in plants and animals by targeting viruses [1,2]. Conversely, miRNAs are produced by viruses [3]. Recent studies have demonstrated that the expression of viral miRNAs is associated with the ability of the virus to infect a host [2,4]. Additionally, studies have reported that viral miRNAs are associated with human diseases [2-8]. For example, the Epstein-Barr virus, hepatitis B and C viruses, and human papillomavirus are highly associated with gastric and nasopharyngeal carcinoma, liver cancer, and cervical cancer, respectively.

Recently, several approaches have been developed for computationally identifying miRNA precursors (pre-miRNAs) [9-15]. Various classifiers, such as the support vector machine (SVM), random forest, relaxed variable kernel density estimator (RVKDE), and bootstrap aggregating, were applied while different features generated from sequences and secondary structural information were employed. The characteristics of the approaches are summarized in Table 1.

**Table 1:** Characteristics of tools for identifying pre-miRNAs.

Tool	Classifier	Used features	*SN (%)*	*SP (%)*	References
**Triplet-SVM**	SVM	Each hairpin is encoded as a set of 32 triplet elements	93.3	88.1	Xue et al. [9]

**MiPred**	Random forest	32 Triplet-SVM features and a minimum of the free energy of the secondary structure	89.3	93.2	Jiang et al. [10]

**miPred**	SVM	17 primary sequencing features, 5 secondary structural features, and 7 normalized features	84.5	97.9	Ng and Mishra [11]

**miR-KDE**	RVKDE	29 miPred features and 4 stem-loop features	88.9	92.6	Chang et al. [12]

**microPred**	SVM	29 miPred features, 4 RNAfold-related features, 6 Mfold-related features, 7 base-pair-related features, and 2 MFE-related features	83.3	99.0	Batuwita et al. [13]

**MiRenSVM**	SVM	8 triplet structural features, 8 base-pair group features, 16 thermodynamic group features	87.7	98.8	Ding et al. [14]

**miR-BAG**	Naïve Bayes BF Tree SVM	4 mononucleotide features, 16 dinucleotide features, 20 triplet structural features, consecutive paired bases, structural profile scoring, and normalized sequence-based total-pairing features	89.8	91.5	Ashwani Jha et al. [15]

*SN*: sensitivity; *SP*: specificity

Triplet-SVM [9] involves applying an SVM to human data by using features from local, contiguous structure-sequence information for distinguishing the hairpins of real pre-miRNAs from pseudo pre-miRNAs. Each hairpin is encoded as a set of 32 triplet elements. MiPred [10] involves applying a random forest machine learning algorithm to human data by using a hybrid feature, which consists of the 32 features used in Triplet-SVM and the minimum of free energy (MFE) of the secondary structure, and using a *P*-value randomization test to distinguish the real pre-miRNAs from other hairpin sequences with similar stem-loops (pseudo pre-miRNAs). The de novo SVM classifier miPred [11] identifies pre-miRNAs without relying on phylogenetic conservation; 17 primary sequence features, 5 secondary structural features, and 7 normalized features are used in the model. The miR-KDE tool [12] was developed using the novel RVKDE classifier, which exploits local information, and is particularly suitable for predicting species-specific pre-miRNAs. Each hairpin-like sequence is summarized as a 33-dimentional feature vector, including the 29 features used in miPred and 4 stem-loop features. The microPred tool [13] uses effective machine learning methods for classifying human pre-miRNA hairpins from both pseudohairpins and other ncRNAs. Each hairpin is encoded using the 29 features from miPred, 6 RNAfold-related features, 4 Mfold-related features, 7 base-pair features, and 2 MFE-related features. The classification results showed reliability in both sensitivity (*SN*) and specificity (*SP*). The MiRenSVM tool [14] is an ensemble-SVM classification system for detecting miRNA genes, especially those with multiloop secondary structures; 8 triplet structural features, 8 base-pair group features, and 16 thermodynamic group features are considered in feature extractions of hairpin-like sequences. The miR-BAG tool [15] uses a bootstrap-aggregation-based machine learning approach to identify miRNA candidate regions in genomes by using scanning sequences. Comparative analysis results showed that miR-BAG performed more favorable than the previous six tools did. A next-generation sequencing module was combined with miR-BAG to provide high-throughput data analysis. Vir-Mir db [16] is a database for collecting predictive viral miRNA candidate hairpins into a virus genome by using the prediction filters of Srnaloop, sequences and structures, and open reading frames.

Most of the previously developed approaches mainly emphasized identifying pre-miRNAs in human, plants, and other animals. Thus far, a method designed specifically for identifying viral pre-miRNAs has not been developed. Therefore, we collected experimentally validated viral pre-miRNA data and constructed a predictive model by using several sequencing and structural features. This model can assist biological researchers who study virus-host interactions in identifying potential viral miRNAs in experimental sequencing data.

## Materials and methods

### Datasets

The positive dataset was collected from miRBase (Version 19). Two hundred sixty-three pre-miRNAs, including 437 mature miRNAs, from 26 virus species were collected as the positive dataset. The negative dataset consisted of three types of sequences, namely the virus genome, human pre-miRNAs, and Pseudo-8494. The virus genome dataset was composed of 789 randomly selected fragments with lengths of 120 bps in the virus genome, and the fragments containing positive data were removed. The human pre-miRNA dataset contained 1600 human pre-miRNAs collected from miRBase. Redundant or highly similar sequences had been removed from the dataset. The negative dataset was obtained from Xue et al. [9] and was used in miPred, miR-KDE, and other tools. We named this benchmark negative dataset "Pseudo-8494" because it was composed of 8494 fragments from the coding regions of human chromosome 19.

### Feature extraction and selection

The SVM and random forest classification methods were applied to develop predictive models for viral-pre-RNA identification. The minimal free-energy and base-pair-related information were obtained using the RNAfold of the Vienna RNA package [17]. Fifty-four features were selected from previous research [9,11,13], and the feature score (*F-score*) [14] was used to evaluate the discriminative power of each feature. The features used in our model are described in the following paragraphs.

#### 32 triplet elements

The local contiguous triplet-structure composition was defined as Triplet-SVM [9]. For the predicted secondary structure, an opening parenthesis "(" or a closing parenthesis ")" and a dot "." were used to denote paired and unpaired nucleotides. Generally, the opening parenthesis "("represents a paired nucleotide located near the 5′-end that can be paired with another nucleotide at the 3′-end, which is denoted by the closing parenthesis ")". This study used "(" for both situations. For any three adjacent nucleotides, there are eight (2^3^) possible triple-structure compositions: "(((", "((.", "(.(", ".((", "(..", ".(.", "..(", and "...". Considering the middle nucleotide, there are 32 (4 × 8) possible structure-sequence combinations, which are denoted as "C(((", "A.((", etc.

#### 4 sequential features

The GC content ratio (*%G+C*), sequence length, hairpin length, loop length in the RNA sequence, and secondary structure were used in the model.

#### 8 thermodynamic features

The *dP*, *dG*, *zP*, *zG*, *MFEI_1_*, and *MFEI_2 _*features were chosen from miPred [11] and the *MFEI_3 _*and *MFEI_4 _*features were selected from microPred [13]. The feature *dP *measures the total number of base pairs present in the RNA secondary structure *S *divided by the length *L *in nucleotides; *dG *is the ratio of the MFE to *L*, which measures the thermodynamic stability of the RNA structure *S*. The features *zP *and *zG *are the normalized variants of *dP *and *dG*; for each original sequence, 1000 random sequences were generated. The feature *MFEI_1 _*is the ratio of *dG *to %G+C; *MFEI_2 _*is the ratio of *dG *to the number of stems *S*, which are structural motifs containing more than three contiguous base pairs; *MFEI_3 _*is the ratio of *dG *to the number of loops in the secondary structure; and *MFEI_4 _*is the ratio of the MFE to the total number of base pairs in the secondary structure.

#### 8 base-pair-related features

The features *|A-U|/L*, *|G-C|/L*, *|G-U|/L*, *%(A-U)/n_stems*, *%(A-U)/n_stems*, *%(A-U)/n_stems*, *consecutive base-pairs (ConsecBP)*, and *Avg_BP_stem *were chosen and introduced in microPred [13], where |X-Y| is the number of (X-Y) base pairs in the secondary structure, (X-Y) ∈ {(A-U), (G-C), (G-U)}. The feature *ConsecBP *represents the longest pairing stretch observed in a given structural sequence, and *Avg_BP_stem *is the ratio of the total number of base pairs to the number of stems in the secondary structure, where a stem is a structural motif containing more than three contiguous stack of base pairs as defined in miPred.

#### 2 RNAfold-related features

The frequency of the MFE structure (*Freq*) and the structural diversity (*Diversity*) were included. These features were generated using the RNAfold program [17] with the "*-p*" option at 37 °C, which calculates the partition function and the base paring probability matrix according to the algorithms proposed in [18].

### Classification and performance evaluation

SVM is a machine learning approach for solving classification and regression problems. It constructs a set of hyperplanes in a high- or infinite-dimensional space and has been widely applied to biological sequence classification. Random forest is a nonparametric tree-based ensemble method that is broadly applied in machine learning and can account for interactions and correlations among features. It constructs multiple decision trees during training time and outputs the class that is the mode of the classes output by individual trees. Here, LIBSVM [19] and random forest approaches [20] were adopted to develop the predictive models for viral pre-miRNA identification.

Fivefold cross validation was applied in a performance evaluation of the predictive models. The *SN*, *SP*, precision (*PRE*), accuracy (*ACC*), balanced ACC, and Matthew's correlation coefficient (*MCC*) were used to measure the classification performance and were defined as follows: *SN = TP/(TP + FN)*; *SP = TN/(TN + FP)*; *PRE = TP/(TP + FP)*; balanced ACC = (*SN *+ *SP*)/2; *ACC = (TP + TN)/(TP + FP + TN + FN)*; and $\text{MCC}=\frac{\text{TP}\times \text{TN}-\text{FP}\times \text{FN}}{\sqrt{\text{(TP}+\text{FN)}\times \text{(TP}+\text{FP)}\times \text{(TN}+\text{FP)}\times \text{(TN}+\text{FN)}}}$, where *TP*, *FP*, *TN*, and *FN *are the numbers of true positives, false positives, true negatives, and false negatives [21], respectively. The *MCC *value is between -1 and 1, where 0 is a completely random prediction, 1 is a perfect prediction, and -1 is a perfectly inverse correlation.

## Results

### Classification results of the SVM and random forest models using different features

Table 2 shows the feature scores of the 54 features as sorted by *F-score *in descending order. The features with the highest *F-scores*, namely 1.09, 1.08, and 1.04, were "*G(((*", "*C(((*", and "*G((*.", respectively. The performance results from the fivefold cross validation of the SVM and random forest models conducted using different negative datasets are shown in Tables 3 and 4. The classification results showed that the SVM model had superior performance when applied to the Pseudo-8494 and human pre-miRNA datasets and that random forest had superior performance when applied to the virus genome dataset.

**Table 2:** *F-scores *of the 54 features.

Feature	***F-score***	Feature	***F-score***	Feature	***F-score***
***G(((***	1.09	*dP*	0.83	*MFEI_2_*	0.63
***C(((***	1.08	*%(G-C)/stems*	0.81	*MFEI_3_*	0.63
***G((*.**	1.04	*G(.(*	0.80	*Avg_BP_Stem*	0.61
***U(((***	1.03	*|A-U|/L*	0.78	*|G-U|/L*	0.60
***A(((***	1.01	*Diversity*	0.78	*G.(*.	0.58
***C(.(***	1.01	*C.((*	0.77	*C..*.	0.58
***U.((***	1	*|G-C|/L*	0.76	*U.(*.	0.57
***U((*.**	1	*U(.(*	0.75	*zP*	0.56
***A.((***	1	*MFEI_1_*	0.72	*A.(*.	0.54
***C((*.**	0.99	*Frequency*	0.71	*MFEI_4_*	0.51
***G(.*.**	0.97	*U..*.	0.71	*U(.*.	0.48
***G.((***	0.97	*A..(*	0.70	*zG*	0.47
***A(.*.**	0.96	*%(A-U)/stems*	0.69	*hairpin length*	0.43
***A(.(***	0.95	*dG*	0.66	*G..(*	0.43
***C.(*.**	0.94	*%(G-U)/stems*	0.66	*G..*.	0.41
***ConsecBP***	0.94	*Loop length*	0.66	*C..(*	0.37
***A((*.**	0.91	*C(.*.	0.66	*sequence length*	0.34
***U..(***	0.87	*%G+C*	0.65	*A..*.	0.32

**Table 3:** Classification results of the SVM model.

Negative dataset	*TP*	*TN*	*FP*	*FN*	*SN*	*SP*	*ACC*
Virus genome	213	661	128	50	80.98%	83.77%	83.07%

Pseudo-8494	202	8403	91	61	76.80%	98.92%	98.26%

Human pre-miRNA	204	1498	102	59	77.56%	93.62%	91.35%

**Table 4:** Classification results of the random forest model.

Negative dataset	*TP*	*TN*	*FP*	*FN*	*SN*	*SP*	*ACC*
Virus genome	215	669	120	48	81.74%	84.79%	84.03%

Pseudo-8494	198	8306	188	65	75.28%	97.78%	97.11%

Human pre-miRNA	203	1464	136	60	77.18%	91.50%	89.47%

Additionally, a compact model was constructed using the features with *F-scores *higher than 0.6; 40 features were used in this model. The classification results for the SVM and random forest models are shown in Tables 5 and 6. The results showed that the performance of both models increased after feature selection. The performance of the SVM model was superior to that of the random forest model for all datasets, achieving ACC values of 86.02%, 97.85%, and 90.23% when applied to the negative virus genome, Pseudo-8494, and human pre-miRNA datasets, respectively. Therefore, the SVM model was chosen as our final predictor for viral pre-miRNA identification.

**Table 5:** Classification results of the SVM model using the 40 features with the highest *F-scores*.

Negative dataset	*TP*	*TN*	*FP*	*FN*	*SN*	*SP*	*ACC*
Virus genome	224	690	99	39	85.17%	87.45%	86.88%

Pseudo-8494	207	8389	105	56	78.70%	98.76%	98.16%

Human pre-miRNA	211	1487	113	52	80.22%	92.93%	91.14%

**Table 6:** Classification results of the random forest model using the 40 features with the highest *F-scores*.

Negative dataset	*TP*	*TN*	*FP*	*FN*	*SN*	*SP*	*ACC*
Virus genome	219	686	103	44	83.26%	86.94%	86.02%

Pseudo-8494	201	8368	126	62	76.42%	98.51%	97.85%

Human pre-miRNA	208	1473	127	55	79.08%	92.06%	90.23%

### Comparison with previous studies using an independent dataset

For a comparison of our approach with previously proposed approaches, 63 viral pre-miRNAs from a positive dataset and 189 sequences from a virus genome dataset were collected as an independent testing dataset, and our model for the comparison was constructed using the remaining data. Seven tools, namely Triplet-SVM, MiPred, miPred, miR-KDE, microPred, MiRenSVM, and miR-BAG, were used for the comparison. The classification results are shown in Table 7. The results showed that miPred had the highest *SP *(93.65%), *ACC *(86.9%), and *MCC *(0.63). Our approach had the highest *SN *(79.36%) as well as the highest balanced ACC (83.06%), which is calculated by considering the inflation of performance estimates caused by the use of an imbalanced dataset. Some testing data could be used from previous approaches when constructing the model, potentially resulting in an increase of the prediction performance.

**Table 7:** Performance comparison with previous studies using a partial dataset.

Tool	Positive dataset/negative dataset	*TP*	*TN*	*FP*	*FN*	*SN*	*SP*	*ACC*	*Balanced ACC*	*MCC*
**Triplet-SVM**		44	171	18	19	69.84%	90.47%	85.32%	80.15%	0.61
		
**MiPred**		41	175	14	22	65.07%	92.59%	85.71%	78.83%	0.60
		
**miPred**		42	177	12	21	66.66%	93.65%	86.90%	80.16%	0.64
		
**miR-KDE**	63/189	39	176	13	24	61.90%	93.18%	85.31%	77.51%	0.59
		
**microPred**		48	159	30	15	76.54%	84.12%	82.14%	80.16%	0.56
		
**MiRenSVM**		45	161	28	18	71.45%	85.21%	81.75%	78.30%	0.54
		
**miR-BAG**		46	166	23	17	73.01%	87.83%	84.13%	80.42%	0.59
		
**Our approach**		50	164	25	13	79.36%	86.77%	84.92%	83.06%	0.63

In addition to the use of the partial dataset for independent testing, 32 newly released virus pre-miRNAs from miRBase (Version 20) were collected as the positive data, and 96 randomly selected fragments from the virus genome were generated as the negative dataset. The classification results are shown in Table 8. The results showed that Triplet-SVM had the highest specificity (91.67%) and *ACC *(85.94%). Our model, ViralmiR, had the highest sensitivity (78.13%), *ACC *(85.94%), balanced ACC (83.33%), and *MCC *(0.64). The results showed that ViralmiR exhibited favorable performance in viral pre-miRNA identification and was superior to related predictors.

**Table 8:** Performance comparison with previous studies using newly released data from miRBase.

Tool	Positive dataset/negative dataset	*TP*	*TN*	*FP*	*FN*	*SN*	*SP*	*ACC*	*Balanced ACC*	*MCC*
**Triplet-SVM**		22	88	8	10	68.75%	91.67%	85.94%	80.21%	0.62
		
**MiPred**		20	79	17	12	62.50%	82.29%	77.34%	72.40%	0.43
		
**miPred**		24	85	11	8	75.00%	88.54%	85.16%	81.77%	0.62
		
**miR-KDE**	32/96	23	81	15	9	71.88%	84.38%	81.25%	78.13%	0.53
		
**microPred**		23	86	10	9	71.88%	89.58%	85.16%	80.73%	0.61
		
**MiRenSVM**		19	81	15	13	59.38%	84.38%	78.13%	71.88%	0.43
		
**miR-BAG**		22	82	14	10	68.75%	85.42%	81.25%	77.08%	0.52
		
**ViralmiR**		25	85	11	7	78.13%	88.54%	85.94%	83.33%	0.64

### Web interface

**Figure 1:**
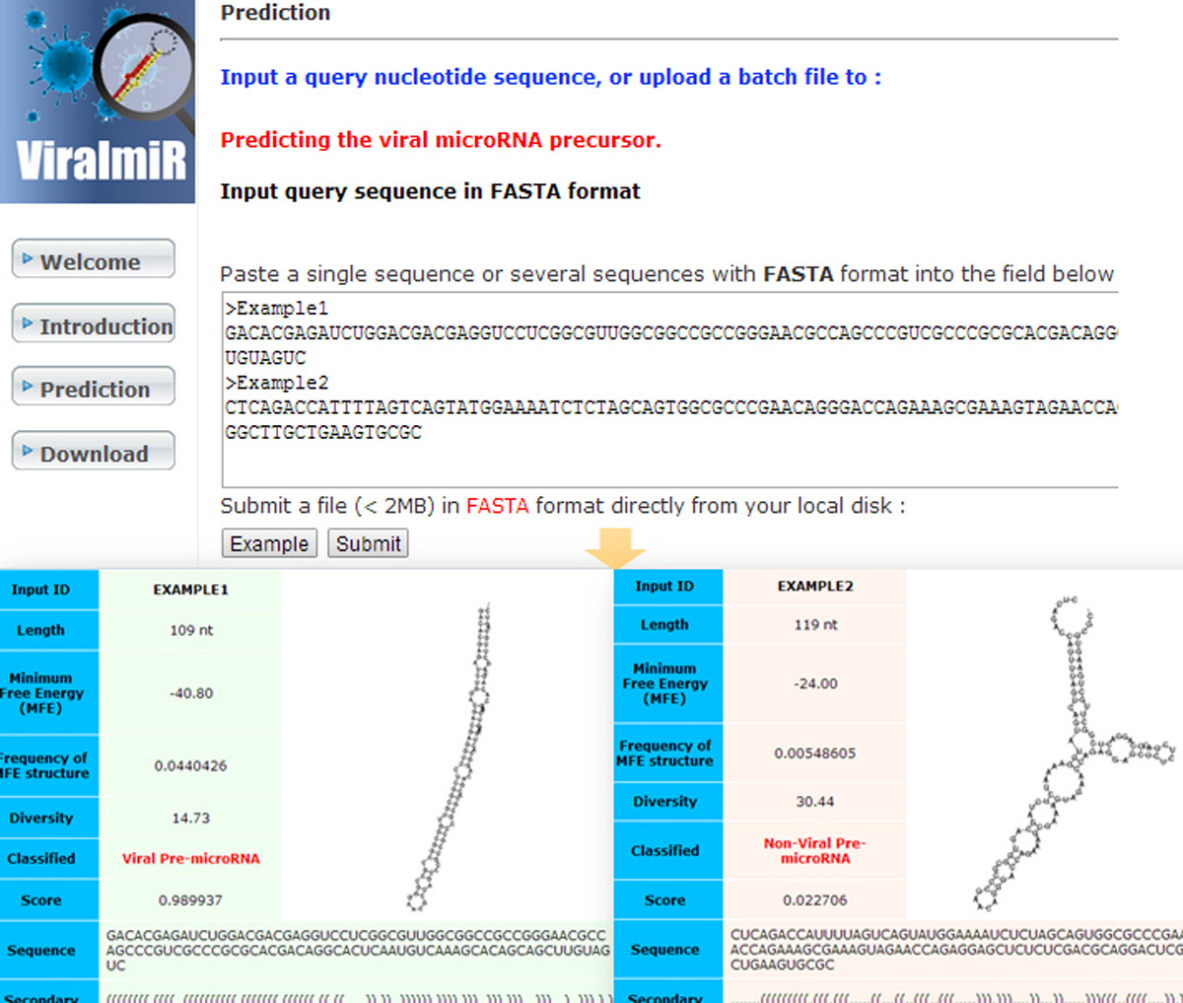
**Web interface of ViralmiR**.

A ViralmiR web interface was developed for identifying viral pre-miRNAs in RNA sequences. As shown in Figure 1, the ViralmiR web page provides a user-friendly interface and information related to predictive results. Users of the website can submit a sequence in the FASTA format to identify potential viral pre-miRNA. The positive dataset and three negative datasets used in this study are also provided on the website. The web server is available at http://csb.cse.yzu.edu.tw/viralmir/.

## Discussion and conclusion

As shown in Table 2 the features with high *F-scores *are related to triplet elements, with "G(((" and "C(((" being the features having the highest *F-scores*. Other base-pair- and thermodynamic-related features, such as *ConsecBP*, *dP*, and *%(G-C)/stems*, also had high *F-score *values. The results showed that the G-C base-pair-related features play vital roles in viral pre-miRNA identification. In our analysis pipeline, the secondary structure for sequences was derived using RNAfold. However, in many instances, the structure predicted using the MFE may not resemble the real structure, and, thus, the predicted structure of the viral pre-miRNAs could not be formed as a hairpin-like shape, affecting the performance of the predictive models. An examination of our positive dataset showed that only 219 of the 263 viral pre-miRNAs could be formed into hairpin-like shapes by using the MFE. The other pre-miRNAs were formed in other shapes. Table 9 shows the number of hairpin-like and non-hairpin-like shapes in true-positive and false-negative predictions. The results show that most sequences (more than 93%) of true positive prediction were formed in hairpin-like shapes and most sequences (more than 77%) of false negative prediction were not formed in hairpin-like shapes in SVM model. e similar situations present in random forest model, showing that the prediction performance is highly associated with structural prediction. Therefore, further analysis of various folding parameters and window sizes is warranted to facilitate obtaining a more suitable parameter combination for predicting the secondary structure of viral pre-miRNAs.

**Table 9:** Number of hairpin-like shapes and non-hairpin-like shapes in prediction results

	True-positive predictions		False-negative predictions	
	
	Hairpin-like shapes	Non-hairpin-like shapes	Hairpin-like shapes	Non-hairpin-like Shapes
SVM model	210 (93%)	14 (7%)	9 (23%)	30 (77%)
Random forest model	208 (95%)	11 (5%)	11 (25%)	33 (75%)

A tool for predicting viral pre-miRNAs in sequences can benefit biomedical researchers who study interactions between viral miRNAs and host genes. In this study, we present a virus-specific pre-miRNA prediction model, ViralmiR, based on sequence and RNA secondary-structure information. ViralmiR achieved a balanced ACC higher than 83%, which is superior to that of previously developed predictors. The easy-to-use ViralmiR web interface has been provided as a helpful resource for researchers to use in analyzing and deciphering virus-host interactions.

## Availability and requirements

The ViralmiR system is freely available at http://csb.cse.yzu.edu.tw/viralmir/.

## Competing interests

The authors declare that there are no competing interests.

## Authors' contributions

TYL and THC conceived and supervised the study and drafted the manuscript. KYH and YCT were responsible for the design, computational analyses, and implementation of the system. All authors read and approved the final manuscript.

## Declarations

The authors approved the submission of this paper to BMC Bioinformatics for publication. The payment of publishing charges to BioMed Central for this article was supported by Ministry of Science and Technology of the Republic of China, No. MOST 103-2221-E-038-013-MY2, 103-2221-E-155-020-MY3 and 103-2633-E-155 -002.

This article has been published as part of *BMC Bioinformatics *Volume 16 Supplement 1, 2015: Selected articles from the Thirteenth Asia Pacific Bioinformatics Conference (APBC 2015): Bioinformatics. The full contents of the supplement are available online at http://www.biomedcentral.com/bmcbioinformatics/supplements/16/S1
